# Arachidonate 15-Lipoxygenase Enzyme Products Increase Platelet Aggregation and Thrombin Generation

**DOI:** 10.1371/journal.pone.0088546

**Published:** 2014-02-12

**Authors:** Carolina Vijil, Cecilia Hermansson, Anders Jeppsson, Göran Bergström, Lillemor Mattsson Hultén

**Affiliations:** 1 Department of Clinical Chemistry, Sahlgrenska University Hospital, Gothenburg, Sweden; 2 Department of Molecular and Clinical Medicine, Institute of Medicine, Sahlgrenska Academy, University of Gothenburg, Gothenburg, Sweden; 3 Department of Cardiothoracic Surgery, Sahlgrenska University Hospital, Gothenburg, Sweden; Brigham and Women's Hospital, Harvard Medical School, United States of America

## Abstract

Atherosclerotic cardiovascular diseases are the leading causes of morbidity and mortality worldwide. We have previously shown that arachidonate 15-lipoxygenase B (ALOX15B) is highly expressed in atherosclerotic carotid plaques, and elucidation of mechanisms downstream of activated lipoxygenases may be relevant to our understanding of the genesis of atherosclerotic diseases. We examined 120 carotid plaques from patients with symptomatic carotid artery stenosis and showed that the extent of ALOX15B staining was significantly increased in carotid plaques with thrombosis. Impedance aggregometry analyses showed that the ALOX15B enzyme products 15-HETE and 15-HPETE increased platelet aggregation. By using a calibrated automatic thrombin assay, we showed that the ALOX15B products also increased both peak levels of thrombin and the total endogenous thrombin potential. Moreover, platelet aggregation was increased by addition of cell lysates from ischemic human macrophages, whereas platelet aggregation was reduced after knockdown of ALOX15B in human macrophages. Our data show that ALOX15B expression in human carotid plaques is associated with thrombus formation and that enzyme products of ALOX15B increase platelet aggregation and thrombin generation. We therefore propose that activated ALOX15B in macrophages may play a role in the induction of atherothrombotic events by increasing platelet aggregation and thrombin generation.

## Introduction

Atherosclerotic lesions are common in the carotid artery, but only a minority of the plaques ever causes cerebrovascular ischemic events. Symptomatic carotid atherosclerotic plaques and rupture-prone vulnerable plaques have histopathological features such as inflammatory monocytes/macrophages, cell infiltration and a thin fibrous cap leading to hemorrhage. Chronic inflammation is evident at every stage from initiation to progression and eventually to plaque rupture and thrombosis [1]. We previously showed that arachidonate 15-lipoxygenase type B (ALOX15B) is locally enriched within symptomatic atherosclerotic plaques, especially in regions with high macrophage density [2]. Human ALOX15B catalyzes the formation of 15-hydroxy eicosatetraenoic acid (15-HETE) and 15-hydroperoxy eicosatetraenoic acid (15-HPETE) from arachidonic acid [3]. Macrophages play integral roles in atherosclerosis and are key regulators of the lipid-driven proinflammatory responses that promote atherosclerosis [4], and proinflammatory activation of macrophages leads to activation of ALOX15B and increased production of 15-HETE [5]. Our previous data also show that reduction of ALOX15B decreases inflammation and lipid accumulation, both in human primary macrophages and in mice, suggesting an active proinflammatory and proatherogenic role of ALOX15B [6].

Inflammation in the vascular wall and thrombosis are linked processes, and cellular activation with production of proinflammatory molecules may also be prothrombotic [7]. It is well known that arachidonic acid and the cyclooxygenase enzyme product thromboxane A_2_ increase platelet aggregation [8] but little is known about the effects of arachidonate-lipoxygenase products on platelet aggregation and thrombus formation. However, a recent biomarker study found increased HETE levels in the plasma of ischemic stroke patients [9], suggesting that ALOX15B products may play a role in thrombosis.

Here, we studied carotid plaques from patients who suffered an ischemic event caused by carotid atherosclerosis and investigated if ALOX15B expression in these carotid plaques is associated with thrombus formation. We also investigated how the ALOX15B enzyme products 15-HETE and 15-HPETE affect platelet aggregation.

## Materials and Methods

### Human carotid endarterectomies

Carotid endarterectomies from 120 patients with high-grade symptomatic carotid artery stenosis (≥70% stenosis according to European Carotid Surgery Trial criteria [10]) were obtained from the Göteborg and Umeå Vascular Study biobank (http://www.wlab.gu.se/bergstrom/guvasc/). Patient characteristics are shown in Table 1.

**Table 1:** Patient and plaque characteristics (n = 120).

Characteristic	n (%) or median
Sex	
Female	34 (28%)
Male	86 (72%)
Age, years	69
Type of event	
Stroke	62 (52%)
Transient ischemic attack	31 (26%)
Amaurosis fugax	27 (22%)
Plaque characteristics	
AHA class III/IV/V/VI	5/38/18/59
No thrombosis/thrombosis	74/46
Days since last clinical event	84 (72)
Diabetes	37 (31%)
Hypertension	81 (68%)
Previous myocardial infarction	23 (19%)
Peripheral artery disease	15 (13%)
Current smoker	28 (23%)
Statin treatment	85 (71%)

AHA class, classification of plaques according to American Heart Association.

Immunohistochemical staining was done with rabbit polyclonal anti-ALOX15B (1∶500, Oxford Biomedical Research, Oxford, MI, USA), mouse monoclonal anti-CD68 (1∶500, Leica Novocastra, Kista, Sweden), CD42 mouse monoclonal antibody (1∶200, Dako, Glostrup, Denmark). Detection was performed with Mach3 kit and Vulcan Fast Red (Biocare Medical, CA, USA). Stained sections were digitalized using a Zeiss Mirax Scanner (Zeiss, Jena, Germany). Digital images were analyzed using the BioPix software (BioPix AB, Gothenburg, Sweden). The extent of immunohistochemical staining is expressed as a percentage of the stained area of the total section area.

Thrombus formation was classified using the following criteria: rupture of the fibrous cap with clear communication between the necrotic core and the lumen and adjacent surface thrombus (Figure S1A–C).

Histopathological classification of plaques was made on deparaffinized serial sections stained with Mayers hematoxylin end eosin (Histolab, Gothenburg, Sweden) according to the American Heart Association (AHA) classification [11] using a size Axio Imager M1 microscope (Göttingen, Germany).

### Ethics statement

The study protocol was approved by the Ethical Committee of the University of Gothenburg (Dnr.404–09) and all subjects gave written informed consent.

### Primary macrophages

Buffy coats were obtained from 8 healthy adult volunteer blood donors at Kungälv Hospital, Sweden, and samples were de-identified before handling. Human mononuclear cells were isolated by centrifugation in a discontinuous gradient of Ficoll-Paque (GE Healthcare). Cells were seeded in Macrophage-SFM medium (Gibco) containing granulocyte macrophage colony stimulating factor (GM-CSF). After 3 days, the medium was changed to RPMI 1640 medium without GM-CSF and cells were cultured for 7 days before further experiments. To test the effect of ischemia, cells from 4 blood donors were incubated in 21% or 1% oxygen for 24 hours. To test the effect of ALOX15B knockdown, cells from a further 4 blood donors were incubated in 1% oxygen and transfected with 20 nmol/L ALOX15B siRNA (Qiagen, SI03076206) or nonsilencing control siRNA (Qiagen, 1027280) in HiPerFect transfection reagent (Qiagen) according to the manufacturer's recommendations. Cells were washed after 24 hours, siRNA was added again and cells were incubated at 1% oxygen for 24 hours before extraction of RNA.

Total RNA was isolated with the RNeasy kit (Qiagen). Expression of human ALOX15B mRNA was determined and normalized to β-actin mRNA expression using quantitative real-time PCR. The reverse transcription reaction was set up using a cDNA reverse transcription kit (#4368814) and performed with a Gene Amp PCR system 9700 (Applied Biosystems). Real-time PCR amplification was set up using TaqMan gene expression assays for human ALOX15B (Hs00153988_m1), human actin B (Hs99999903_m1) in combination with Universal PCR master mix (#4324018) and performed for 50 cycles on an ABI PRISM 7700 sequence detection system (Applied Biosystems).

Cell lysates for platelet aggregation and tissue factor measurements were prepared by adding lysis buffer containing 0.15 mmol/l NaCl, 10 mmol/L Tris-HCl, 2 mmol/L EDTA and 1% TritonX-100 with protease inhibitors (Complete Mini, Roche Diagnostics). Cell lysates for measurement of thrombin generation and 15-HETE analysis were prepared by adding cold PBS supplemented with protease inhibitors (Complete Mini) to the macrophages, cell were lysed by freezing at -20°C and thawing four times before analysis.

### Measurement of 15-HETE and tissue factor

15-HETE was analyzed in cell lysates by 15-HETE ELISA kit (ab133035, Abcam, Cambridge, UK). Tissue factor was analyzed in cell lysates by Human Coagulation Factor III/Tissue Factor Quantikine ELISA kit (R&D Systems Europe; Abingdon, UK).

### Measurement of platelet aggregation

Whole blood platelet aggregation was measured using the Multiplate^®^ Platelet Function Analyzer (Roche Diagnostics, GmbH, Mannheim, Germany) as described [12]. Blood from 4 healthy volunteers at Clinical Chemistry, Sahlgrenska University Hospital, Sweden, was collected in hirudin tubes and samples were de-identified before handling. To measure the effect of HETEs and cell lysates on platelet aggregation, 3 µL 15-HETE or 15-HPETE (Cayman Chemical Company, Ann Arbor, USA; 10 nmol/L dissolved in ethanol), ethanol control or 3 µL lysates from primary macrophages was added to tubes containing 300 µl whole blood and 300 µl 0.9% NaCl together with 1.6 µmol/L thrombin-receptor activating peptide in the Multiplate Impedance Aggregometer. Impedance aggregometry was recorded for 5 minutes.

### Measurement of thrombin generation

The calibrated automated thrombin® (CAT) assay (Thrombinoscope BV, Maastricht, the Netherlands) was used to study the effect of 15-HETE and 15-HPETE on thrombin peak level and the total endogenous thrombin potential as previously described [13], [14]. Thrombin generation was analyzed by adding 10 µL 15-HETE, 15-HPETE (10 nmol/L final concentration dissolved in 2% DMSO), DMSO control or 10 µL cell lysates to 20 µL of a platelet-poor plasma reagent (containing 1 pmol/L tissue factor and 4 µmol/L phospholipids; Thrombinoscope), 70 µL pooled normal plasma (Cryocheck, Precision BioLogic, Dartmouth Canada) and 20 µL fluorogenic substrate (containing calcium chloride solubilized in DMSO, Thrombinoscope) in 96-well plates (Immulon, 2HB clear U-bottom, Thermo Electron, Villebon-sur-Yvette, France). Thrombin generation was measured for 20 min using the microplate fluorometer Fluoroskan Ascent® (Thermolabsystems, Helsinki, Finland), with software version 3.0.0.29 (Thrombinoscope).

### Statistics

Data are plotted as mean and SEM unless stated otherwise. All analyses were performed using GraphPad Prism version 5.01 for Windows (GraphPad Software, San Diego, California USA); a 95% confidence interval was used and P values <0.05 were considered significant. Correlations between groups were determined using non-parametric two tailed correlation (Spearman two tailed correlation). Differences between groups were determined using non-parametric two tailed t-test (Mann-Whitney two tailed t-test) or ANOVA.

## Results

### ALOX15B staining associates with thrombosis in human carotid plaques

Patient and plaque characteristics are shown in Table 1. Of the 120 patients, 62 had previously suffered stroke and 31 had experienced a transient ischemic attack (TIA). Evidence of thrombosis was observed in 46 of the 120 plaques. Classification of the 120 plaques according to the AHA criteria is shown in Table 1.

**Figure 1:**
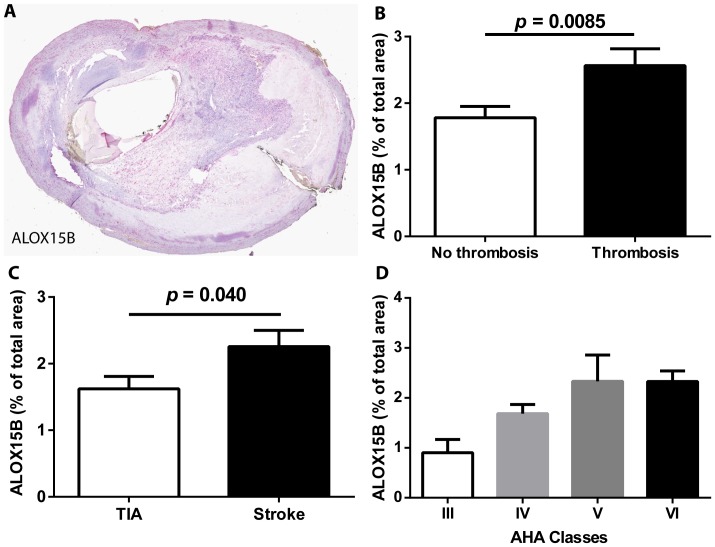
ALOX15B is present in atherosclerotic plaques. Serial sections of atherosclerotic plaques from the carotid artery of patients with symptomatic carotid artery stenosis (n = 120) were stained with antibodies against ALOX15B and counterstained with Mayer's hematoxylin. A representative section is shown in (**A**). (**B**) ALOX15B staining in plaques with thrombosis (n = 46) and without thrombosis (n = 74). (**C**) ALOX15B staining in plaques from patients diagnosed with stroke (n = 62) or TIA (n = 31). (**D**) ALOX15B staining in plaques classified according to AHA classes: III n = 5; IV n = 38; V n = 18; VI n = 59 (AHA class VI indicates complicated lesions with ruptured fibrous cap and/or surface thrombus). Data are mean ± SEM.

To investigate if ALOX15B could play a role in thrombus formation, we analyzed if the ALOX15B staining was higher in carotid plaques with thrombus. Examination of the 120 human carotid plaques showed that the extent of ALOX15B staining correlated with staining for the macrophage marker CD68 (r_s_ = 0.45, P<0.0001), as expected, and was higher in carotid plaques with thrombosis compared with no thrombosis (Figure 1A, B). Furthermore, ALOX15B staining was higher in plaques from patients diagnosed with stroke than in patients diagnosed with TIA (Figure 1C). Further characterization revealed that ALOX15B staining was higher in the most severe lesion phenotypes (AHA class V and IV) with ruptured fibrous cap and/or thrombosis (Figure 1D). These data indicate that ALOX15B is expressed in macrophage-rich areas and associates with thrombosis and complicated lesions.

### ALOX15B enzyme products increase platelet aggregation and thrombin generation

**Figure 2:**
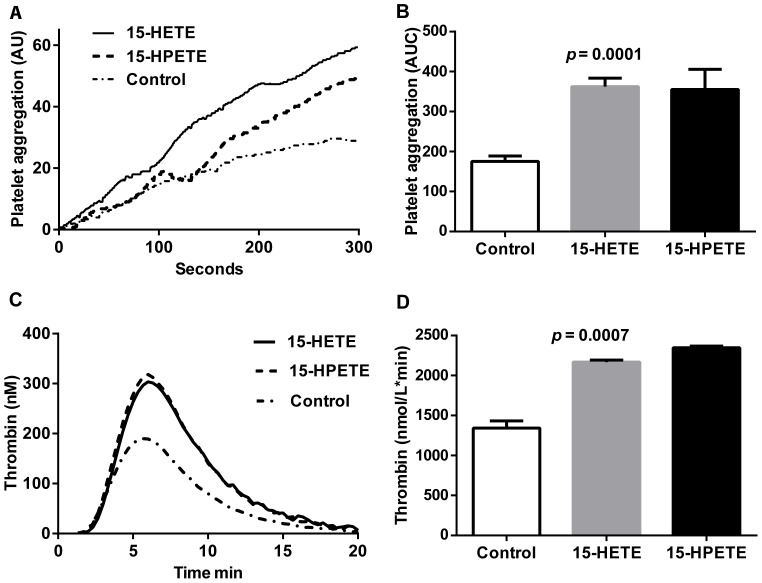
Platelet aggregation and thrombin formation are increased by ALOX15B enzyme products. (A, B) Platelet aggregation was measured in blood (from healthy volunteers) in the presence or absence of 10 nmol/L 15-HETE or 15-HPETE or ethanol control. (**A**) A representative experiment showing platelet aggregation over time. (**B**) Area under the curve (AUC) of platelet aggregation. Data are mean ± SEM from 4 independent experiments (i.e. 4 blood donors) analyzed in duplicate. (**C, D**) Thrombin generation was measured in pooled normal plasma in the presence or absence of 10 nmol/L 15-HETE or 15-HPETE or 2% DMSO control. (**C**) A representative experiment showing thrombin generation curves. (**D**) The total amount of thrombin activity assessed as the area under curve (i.e. the endogenous thrombin potential). Data are mean ± SEM from 4 independent experiments analyzed in duplicate.

To investigate if ALOX15B enzyme products alone can increase platelet activation, blood from healthy volunteers was stimulated with a biological relevant concentration (10 nmol/L) of 15-HETE and 15-HPETE. Both 15-HETE and 15-HPETE increased platelet aggregation in vitro (Figure 2A, B). Because increased thrombin generation is an important factor for platelet aggregation, we also investigated the procoagulant effect of 15-HETE and 15-HPETE using calibrated automated thrombin generation measurements. We showed that addition of 15-HETE and 15-HPETE to serum from healthy volunteers increased both the thrombin peak and the total endogenous thrombin production compared with control (Figure 2C, D). No differences were found for lag time or time to peak in the experiments. Thus, ALOX15B products increased platelet aggregation and thrombin generation, which could lead to detrimental or enhancing effects on blood coagulation and thrombus formation.

**Figure 3:**
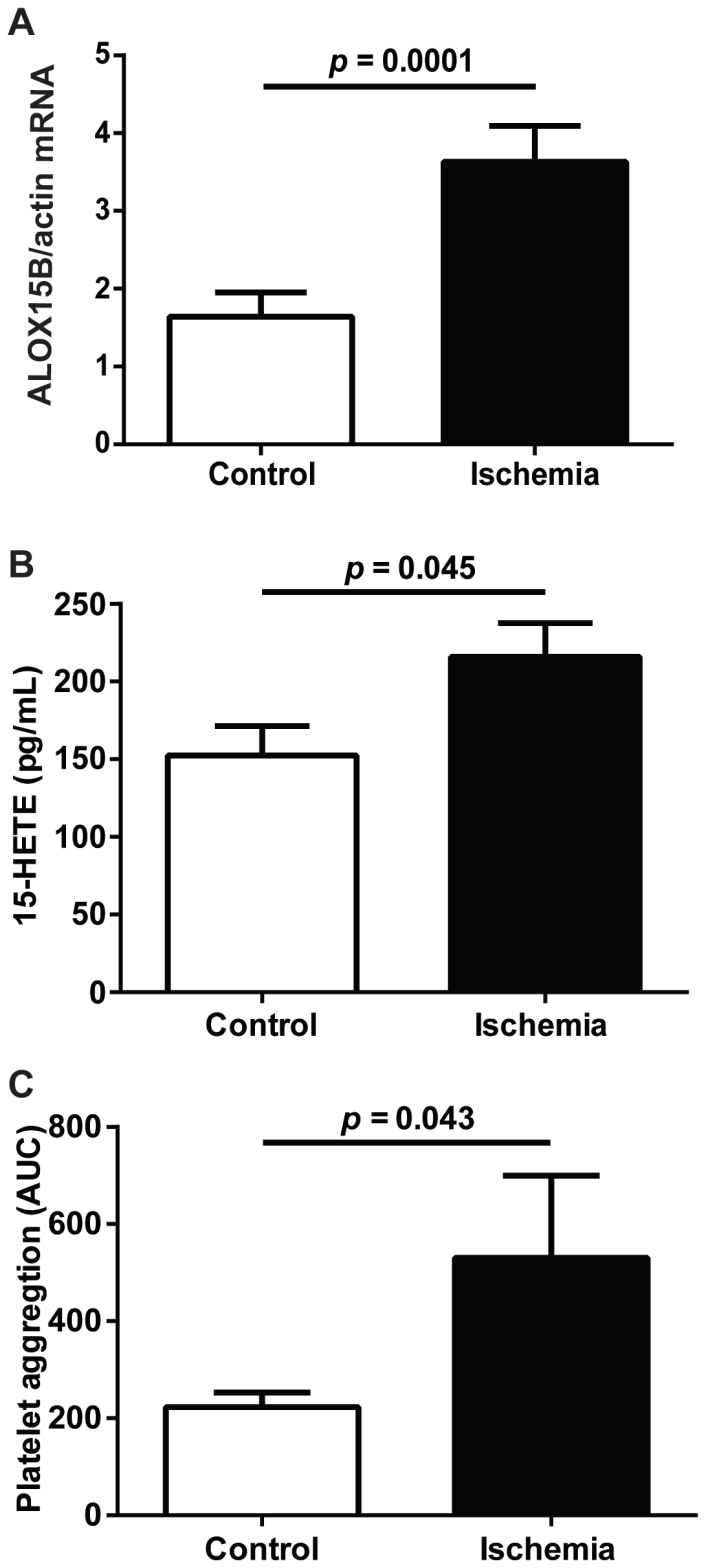
Platelet aggregation is increased by incubation with lysates from human ischemic macrophages. (A) ALOX15B mRNA expression in primary human monocyte-derived macrophages incubated in control (21% oxygen) and ischemic (1% oxygen) conditions. (**B**) 15-HETE production in cell lysates from macrophages incubated in control and ischemic conditions. (**C**) Area under the curve (AUC) of platelet aggregation measured using cell lysates from control and ischemic macrophages. Data are mean ± SEM from 4 independent experiments (i.e. macrophages from 4 blood donors) analyzed in duplicate.

To investigate if ALOX15B enzyme products secreted from ischemic macrophages increase platelet aggregation, we incubated macrophages in a low oxygen concentration to mimic ischemia and activate ALOX15B enzyme activity. We first confirmed our earlier findings that ischemia increases ALOX15B mRNA expression and 15-HETE levels (Figure 3A, B). We also showed that cell lysates from macrophages incubated in ischemic versus normal conditions significantly increased platelet aggregation (Figure 3C) but had no effect on tissue factor levels (Figure S2A).

### ALOX15B knockdown in human macrophages decreases platelet aggregation and thrombin generation

**Figure 4:**
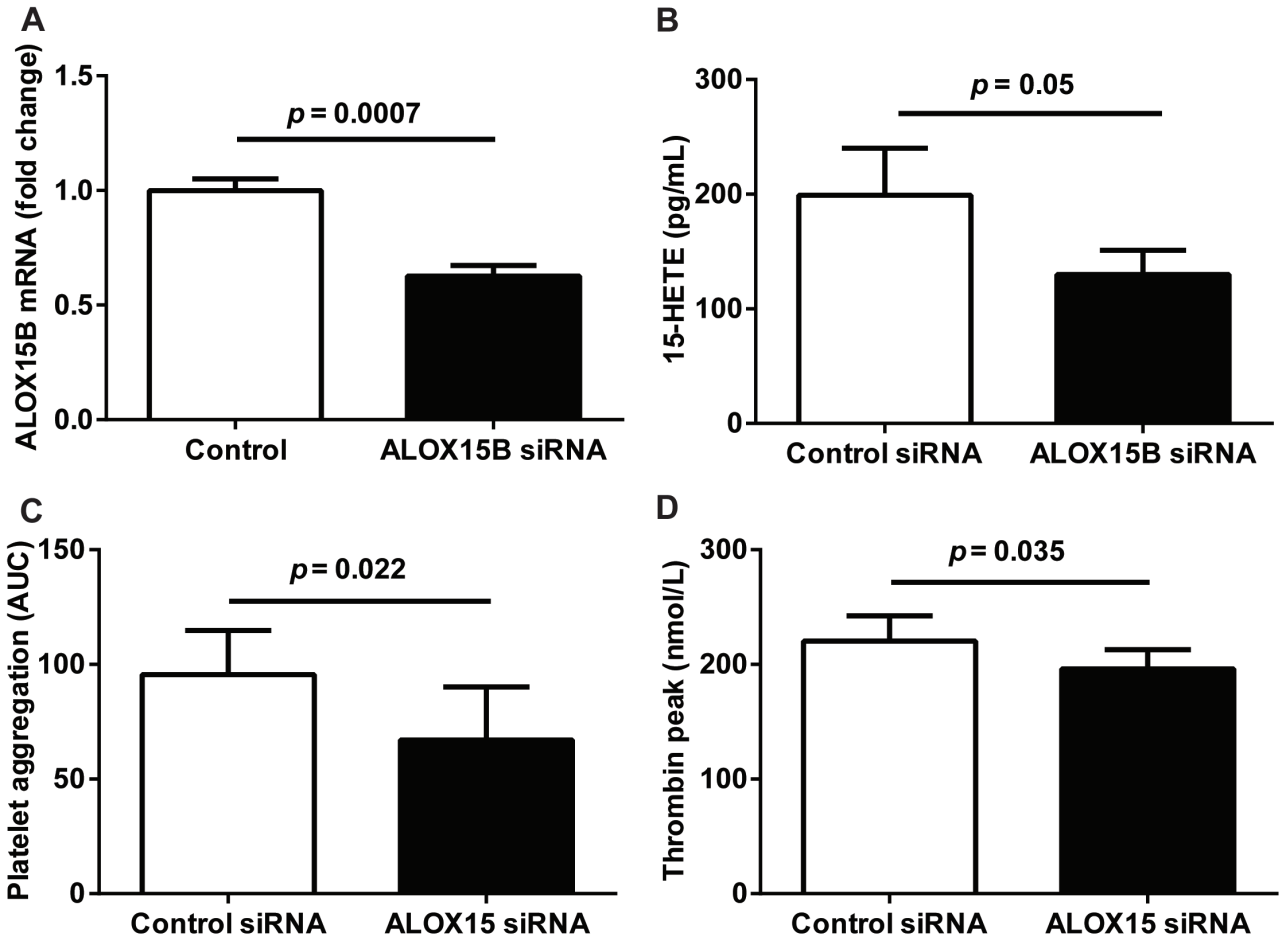
ALOX15B knockdown reduces platelet aggregation and thrombin generation. Primary human monocyte-derived macrophages were transfected with non-silencing control siRNA or ALOX15B siRNA and incubated for 24 hours in ischemia. (A) ALOX15B mRNA expression in macrophages transfected with control or ALOX15B siRNA. (**B**) 15-HETE production in cell lysates from macrophages transfected with control or ALOX15B siRNA. (**C**) Area under the curve (AUC) of platelet aggregation measured using cell lysates from macrophages transfected with control or ALOX15B siRNA. (**D**) Peak thrombin value measured in pooled normal plasma incubated with cell lysates from macrophages transfected with control or ALOX15B siRNA. Data are mean ± SEM from 4 independent experiments (i.e. macrophages from 4 blood donors) analyzed in duplicate.

To specifically investigate the importance of ALOX15B in platelet activation and thrombin generation, we transfected human primary macrophages with control siRNA and ALOX15B siRNA. Knockdown of ALOX15B resulted in a reduction of mRNA levels to 55% of non-silenced levels (Figure 4A) and significantly reduced levels of 15-HETE (Figure 4B). We also showed that ALOX15B knockdown resulted in decreased platelet aggregation (Figure 4C) and thrombin generation (Figure 4D) but had no effect on tissue factor levels (Figure S2B).

## Discussion

The present study showed that ALOX15B expression in human carotid plaques was associated with thrombus formation in the plaque, and high expression of ALOX15B was found in patients diagnosed with stroke. The ALOX15B enzyme products 15-HETE and 15-HPETE increased platelet aggregation and thrombin generation in vitro. Further analysis showed that knockdown of ALOX15B in primary human macrophages decreased 15-HETE production, platelet aggregation and thrombin generation.

It is clear that atherosclerosis is a chronic inflammatory condition that can lead to an acute clinical event by plaque rupture and thrombosis [15]. Pathological studies suggest that the development of thrombus-mediated acute events depends primarily on the composition and vulnerability of a plaque and effects beyond degree of stenosis are likely to play a major role [16]. Vulnerable plaques generally have increased numbers of macrophages that produce various proteases, which degrade extracellular matrix and cause plaque rupture [17]. Rupture frequently occurs in lesions that are rich in macrophages, suggesting that factors contributing to inflammation may also influence thrombosis [16]. We have previously shown that ALOX15B is expressed in macrophage-rich regions [2], and here we extended these findings to demonstrate that ALOX15B is higher in carotid plaques with thrombosis versus no thrombosis and in plaques from patients who had suffered an ischemic stroke compared with TIA. Furthermore, ALOX15B is higher in plaques classified as AHA class V and VI, which represent complicated lesions with ruptured fibrous cap and/or surface thrombus.

Previous studies have reported associations between high ALOX15B expression in carotid lesions and a history of cerebrovascular symptoms [18], suggesting that ALOX15B expression may play a role in mechanisms leading to an ischemic event. A recent biomarker study investigated oxidative changes after ischemic stroke and identified increased HETE levels in the plasma of patients diagnosed with stroke compared with age-matched control subjects [9]. Therefore, we hypothesized that ALOX15B enzyme products may increase platelet activation.

Platelet aggregation measurements are used to predict the occurrence of thrombotic events following percutaneous coronary interventions [19], and increased platelet aggregation has been associated with a 4-fold increased risk of cardiovascular events [20]. Cyclooxygenase activation is known to activate platelet aggregation and thromboxane A_2_ is regarded as one important agonist in this process [21]. The enzyme products 15-HETE and 15-HPETE secreted from human macrophages can also promote platelet aggregation, and may thus be associated with an increased risk of thrombosis. 15-HETE has previously been shown to potentiate platelet aggregation [22], and since this eicosanoid is elevated in pathologic states associated with platelet hyperfunction, including diabetes mellitus and atherosclerosis, an elucidation of its mechanism of action appears relevant to our understanding of the genesis of atherothrombotic vascular disease. Thrombin is an important player in the coagulation system [23], and thrombin generation reflects the functional status of coagulation mechanisms under normal and pathological conditions [24]. Recent insights into coagulation mechanisms show thrombin as the most potent platelet activator and elevated thrombin formation was observed in atherosclerotic carotid plaques [25]. High levels of thrombin generation were associated with an increased risk of acute ischemic stroke [26]. Our results showed that 15-HETE and 15-HPETE increase thrombin generation. Thus, our data demonstrate that the ALOX15B products secreted from ischemic macrophages are prothrombotic substances, which leads us to speculate that patients with increased ALOX15B expression in their carotid arteries may have increased risk of thrombotic events.

Our previous results showed that overexpression of ALOX15B in human macrophages resulted in increased secretion of 15-HETE [27]. Here we show that inhibition of ALOX15B by siRNA decreased levels of 15-HETE and also decreased platelet aggregation and thrombin formation. Importantly, in a mouse model of cerebral ischemia, ALOX12/15 inhibition protected cells against oxidative stress and the ALOX12/15 inhibitor reduced infarct sizes both 24 hours and 14 days post-stroke with improved behavioral parameters [28]. These data suggest that ALOX15B products may play an active role in thrombus formation.

Tissue factor (also known as coagulation factor III) triggers blood coagulation and is expressed in atherosclerotic plaques; it may therefore contribute to thrombus formation after plaque rupture [29]. Here we show that neither ischemia nor ALOX15B knockdown affected tissue factor levels in macrophage lysates, indicating that tissue factor does not explain the changes in platelet aggregation and thrombin generation observed in our macrophage experiments.

In summary, this study shows that ALOX15B staining associates with thrombosis in human carotid plaques, and that platelet aggregation and thrombin formation are increased by ALOX15B enzyme products. We therefore propose that activated ALOX15B in carotid plaques plays a role in the induction of atherothrombotic events by increasing thrombin generation and platelet aggregation.

## Supporting Information

Figure S1 **Immunohistochemical detection of thrombus formation in carotid endarterectomy specimen.** (A) Complicated atherosclerotic plaque with a thin fibrous cap and a plaque rupture (arrows) exposing the underlying necrotic core (NC). Thrombus (T) formation is seen in the region of the plaque rupture. Scale bar 1000 µm. (**B**) Thrombus formation was classified using Haematoxylin & Eosin staining using the following criteria in high-magnification: Rupture of the fibrous cap (arrows) with clear communication between the necrotic core (NC) and the lumen (L) and adjacent surface thrombus (T). Scale bar 200 µm. (**C**) High-magnification on CD42 staining visualizing the thrombus (T) in plaque rupture region (arrows). Scale bar 200 µm.
(TIF)

Figure S2 **Ischemia or ALOX15B knockdown had no effect on tissue factor levels in human macrophages.** (A) Primary human monocyte-derived macrophages from 4 blood donors were cultured for 24 hours in in 21% oxygen (Control) and in 1% oxygen (Ischemia). Tissue Factor was analyzed in cell lysates by using Human Coagulation Factor III/Tissue Factor ELISA kit (R&D Systems Europe Ltd. Abingdon, UK). Data shown are representative of 4 independent experiments analyzed in duplicates; mean ± SEM. (**B**) Human primary macrophages from 4 blood donors transfected with non-silencing control siRNA or ALOX15B siRNA and incubated for 24 hours in ischemia. Tissue Factor was analyzed in cell lysates by using Human Coagulation Factor III/Tissue Factor ELISA kit (R&D Systems Europe Ltd.). Data shown are representative of 4 independent experiments analyzed in duplicates; mean ± SEM.
(TIF)
