# Supplementary figures and images for: Arachidonate 15-Lipoxygenase Enzyme Products Increase Platelet Aggregation and Thrombin Generation
Source: PLoS One. 2014 Feb 12;9(2):e88546. doi: 10.1371/journal.pone.0088546 (PMC3922896; doi:10.1371/journal.pone.0088546)

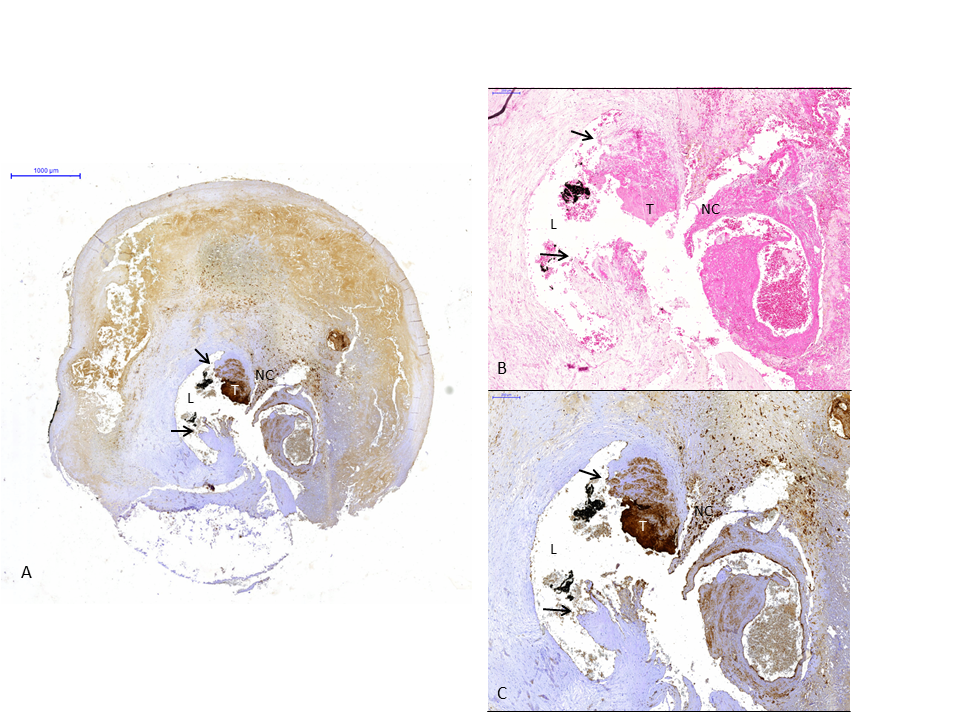

Supplement: Figure S1 — Immunohistochemical detection of thrombus formation in carotid endarterectomy specimen. (A) Complicated atherosclerotic plaque with a thin fibrous cap and a plaque rupture (arrows) exposing the underlying necrotic core (NC). Thrombus (T) formation is seen in the region of the plaque rupture. Scale bar 1000 µm. (B) Thrombus formation was classified using Haematoxylin & Eosin staining using the following criteria in high-magnification: Rupture of the fibrous cap (arrows) with clear communication between the necrotic core (NC) and the lumen (L) and adjacent surface thrombus (T). Scale bar 200 µm. (C) High-magnification on CD42 staining visualizing the thrombus (T) in plaque rupture region (arrows). Scale bar 200 µm. (TIF) [file pone.0088546.s001.tif]

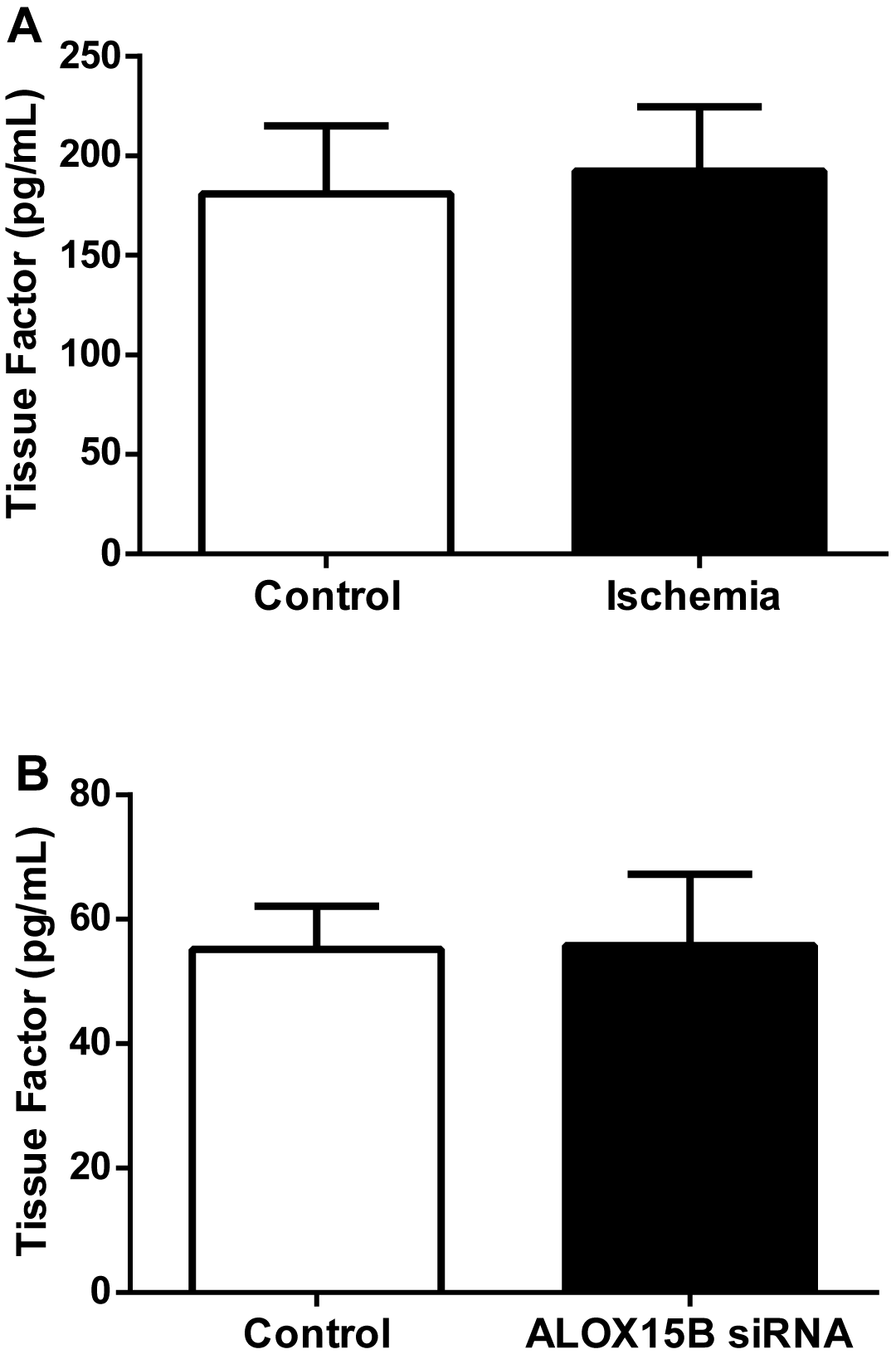

Supplement: Figure S2 — Ischemia or ALOX15B knockdown had no effect on tissue factor levels in human macrophages. (A) Primary human monocyte-derived macrophages from 4 blood donors were cultured for 24 hours in in 21% oxygen (Control) and in 1% oxygen (Ischemia). Tissue Factor was analyzed in cell lysates by using Human Coagulation Factor III/Tissue Factor ELISA kit (R&D Systems Europe Ltd. Abingdon, UK). Data shown are representative of 4 independent experiments analyzed in duplicates; mean ± SEM. (B) Human primary macrophages from 4 blood donors transfected with non-silencing control siRNA or ALOX15B siRNA and incubated for 24 hours in ischemia. Tissue Factor was analyzed in cell lysates by using Human Coagulation Factor III/Tissue Factor ELISA kit (R&D Systems Europe Ltd.). Data shown are representative of 4 independent experiments analyzed in duplicates; mean ± SEM. (TIF) [file pone.0088546.s002.tif]
